# Major Trauma Triage Tool Study (MATTS) expert consensus-derived injury assessment tool

**DOI:** 10.29045/14784726.2024.6.9.1.10

**Published:** 2024-06-01

**Authors:** Gordon Fuller, Nathan Howes, Roderick Mackenzie, Samuel Keating, Janette Turner, Chris Holt, Joshua Miller, Steve Goodacre

**Affiliations:** University of Sheffield ORCID iD: https://orcid.org/0000-0001-8532-3500; Cambridge University Hospitals NHS Foundation Trust; Magpas Air Ambulance ORCID iD: https://orcid.org/0009-0008-7117-7045; Cambridge University Hospitals NHS Foundation Trust; Magpas Air Ambulance ORCID iD: https://orcid.org/0000-0001-6004-0993; University of Sheffield; University of Sheffield ORCID iD: https://orcid.org/0000-0003-3884-7875; University of Sheffield; West Midlands Ambulance Service ORCID iD: https://orcid.org/0000-0003-1990-4029; University of Sheffield ORCID iD: https://orcid.org/0000-0003-0803-8444

**Keywords:** expert consensus, injuries, major trauma, major trauma triage, trauma centres, triage tool

## Abstract

**Introduction:**

Major trauma centre (MTC) care has been associated with improved outcomes for injured patients. English ambulance services and trauma networks currently use a range of triage tools to select patients for bypass to MTCs. A standardised national triage tool may improve triage accuracy, cost-effectiveness and the reproducibility of decision-making.

**Methods:**

We conducted an expert consensus process to derive and develop a major trauma triage tool for use in English trauma networks. A web-based Delphi survey was conducted to identify and confirm candidate triage tool predictors of major trauma. Facilitated roundtable consensus meetings were convened to confirm the proposed triage tool’s purpose, target diagnostic threshold, scope, intended population and structure, as well as the individual triage tool predictors and cut points. Public and patient involvement (PPI) focus groups were held to ensure triage tool acceptability to service users.

**Results:**

The Delphi survey reached consensus on nine triage variables in two domains, from 109 candidate variables after three rounds. Following a review of the relevant evidence during the consensus meetings, iterative rounds of discussion achieved consensus on the following aspects of the triage tool: reference standard, scope, target diagnostic accuracy and intended population. A three-step tool comprising physiology, anatomical injury and clinical judgement domains, with triage variables assessed in parallel, was recommended. The triage tool was received favourably by PPI focus groups.

**Conclusions:**

This paper presents a new expert consensus derived major trauma triage tool with defined purpose, scope, intended population, structure, constituent variables, variable definitions and thresholds. Prospective evaluation is required to determine clinical and cost-effectiveness, acceptability and usability.

## Introduction

Major trauma is a substantial public health problem in England, responsible for 3000 fatalities, 8000 severe disabilities, £400 million of immediate treatment NHS costs and a £3.5 billion loss in economic output each year (Kehoe et al., 2015; National Audit Office, 2010). Major trauma is the leading cause of death in those aged under 40, and its incidence is increasing in the ageing English population (Kehoe et al., 2015). Improvements in the management of major trauma therefore have the potential to greatly improve both health and the economy (Cole et al., 2016; Lockey, 2018).

In 2012, major trauma care in England was reconfigured with the introduction of regional networks, aiming to concentrate seriously injured patients in specialist major trauma centres (MTCs) (Vondy & Willett, 2011). Non-MTC hospitals in England are classified either as trauma units (TUs), equivalent to American College of Surgeons designated Level III or IV trauma centres, or local emergency hospitals (LEHs), equivalent to level V trauma centres, which do not routinely receive trauma patients. MTC care has been associated with improved patient outcomes for severely injured patients (Celso et al., 2006; Moran et al., 2018).

In accordance with the National Institute for Health and Care Excellence (NICE) major trauma service delivery guidelines, pre-hospital triage tools are used within regional trauma networks to identify which patients injured within the catchment areas of TUs and LEHs might benefit from bypass to MTCs (NICE, 2016). Furthermore, relevant to patients injured in both MTC and non-MTC catchment areas, triage tools are also used to inform emergency department (ED) pre-alert calls, facilitating patient reception into resuscitation areas and activation of multi-disciplinary hospital trauma teams. Triage tools must balance under-triage, where patients with major trauma are taken to non-MTCs, delaying time to definitive care and potentially resulting in worse outcomes; and over-triage, where patients conveyed to MTCs do not have major trauma, but contribute to delays in ED access, consume ambulance services, waste MTC resources, reduce TU exposure to injured patients, and separate patients from families for no benefit (Peng & Xiang, 2016).

The NICE major trauma service delivery guidelines highlighted the absence of a national triage tool, and a recent analysis noted that each English ambulance service uses a different tool, with wide variation in the intended population, individual variables and cut points (NICE, 2016). Systematic reviews examining the diagnostic accuracy of international triage tools have suggested that existing tools can be inaccurate (van der Sluijs et al., 2018; van Rein et al., 2017; Voskens et al., 2017), and despite the importance of treating the ‘right person in the right place at the right time’ there has been no consensus on the best triage tool structure, the most appropriate combination of triage variables or the ideal trade-off between under- and over-triage.

The Major Trauma Triage Tool Study (MATTS) is a National Institute of Health and Care Research (NIHR) funded project aiming to develop a standardised national English triage tool. The aim of the current study was to develop an expert consensus-derived major trauma triage tool for future prospective validation during the MATTS project. Specific objectives were to identify candidate triage predictors, define the proposed triage tool’s purpose, scope, structure, intended population and setting, confirm individual triage tool predictors and cut points, and assess acceptability to service users.

## Methods

**Figure 1:**
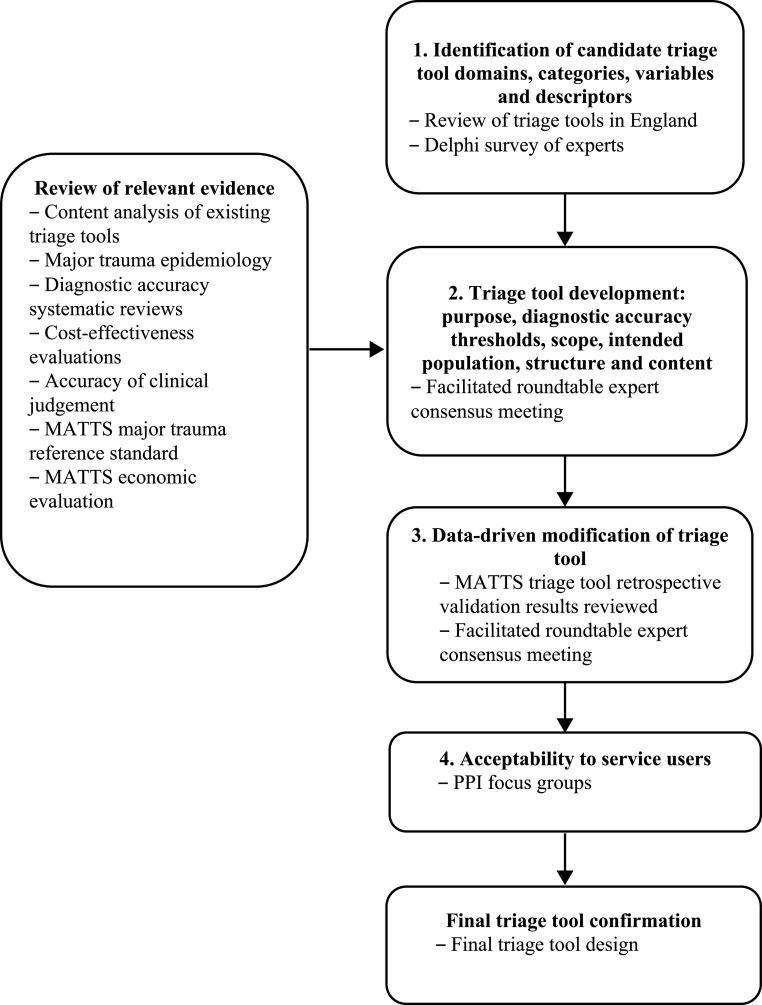
Figure 1. Schematic representation of triage tool development.

The study involved four consecutive phases, as summarised in Figure 1. First, a web-based Delphi survey was conducted to determine candidate variables for a national triage tool. Second, a roundtable consensus meeting was conducted to determine the proposed triage tool’s purpose, target diagnostic threshold, scope, intended population and structure, as well as to confirm the individual triage tool variables and cut points. Third, emerging results from the MATTS retrospective validation study were reviewed by the expert panel to inform triage tool modifications in a further consensus meeting. Fourth, PPI focus groups were held to ensure triage tool acceptability to service users. Study procedures, including sample size determination, followed recommended principles for best practice in developing consensus (Fink et al., 1991; Jones & Hunter, 1995). A study protocol was pre-specified.

The Delphi survey was conducted from January 2016 to January 2017. Purposive sampling was used to recruit a heterogeneous group of experts, representative of clinicians involved across the major trauma clinical pathway, based on their roles as directors or leads of major trauma networks, MTCs, NHS ambulance service trusts or air ambulance services, or their membership of the NHS Major Trauma Clinical Reference Group or Royal College of Emergency Medicine Major Trauma Committee (see Supplementary materials).

The open first round of the classical Delphi approach was modified, with a review of existing English triage tools used to identify possible major trauma predictors (Dalkey et al., 1969). All unique adult triage variables (with or without different cut points or descriptions) were grouped into categories, under domains (physiology, anatomy, mechanism and special circumstances). For example, ‘GCS<14’ and ‘GCS motor score 4 or less’ are different cut points for the ‘Glasgow Coma Scale’ variable, aggregated under the ‘level of consciousness’ category, within the ‘physiology’ domain. Participants were also invited to propose additional novel variables for consideration.

In the second Delphi round, participants were asked whether each domain and category should be included in a national tool, and to rate the appropriateness of each variable. In subsequent rounds, participants revisited domains and categories with moderate agreement, and selected the most appropriate variables (including cut point or descriptor) for each selected category. In each Delphi iteration, participants were provided with the results of the previous round. Consensus for inclusion of each domain and category was defined *a priori* as ≥80%, with exclusion of those with <50% agreement. The number of Delphi rounds was dependent on when group agreement or stability of opinion was achieved on each domain and category.

Following confirmation of included variables and descriptors, further survey rounds were planned to inform the implementation of the triage tool. Participants were asked whether there should be consideration of including the following implementation categories: enhanced care team support, management of immediate clinical risk (e.g. uncontrollable haemorrhage), definition of a time threshold for bypass journeys, and/or inclusion of unstructured clinical judgement. Within each category, possible variables were ranked on a 1 (highly favoured) to 5 (not favoured) Likert scale (Sullivan & Artino, 2013).

Each Delphi round allowed up to four weeks for responses, with email reminders sent after one week. The study would terminate when it was obvious that no further consensus was possible with further iterations. Surveys were designed, pre-piloted and implemented using the SurveyMonkey web application (Momentive Inc., San Mateo, California, USA). Data analysis was performed in Microsoft Excel (Microsoft Corporation, Redmond, USA). No financial incentives were offered, and participants remained anonymous throughout the study.

Second, a one-day facilitated roundtable expert consensus meeting was conducted to finalise triage tool structure and content. Eleven clinicians were purposively sampled to form an expert clinical panel, ensuring that all clinicians relevant to pre-hospital major trauma triage were represented (see Supplementary materials). The meeting was co-chaired by an experienced health service researcher (JT) and a senior emergency medicine academic (SG); and was conducted at the University of Sheffield in September 2019 (Halcomb et al., 2008; Jones & Hunter, 1995).

Introductory sessions were provided to cover: the findings of the preceding Delphi survey, major trauma triage concepts (Peng & Xiang, 2016; Sharma, 2005), major trauma reference standards (Fuller et al., 2021), existing triage tools (van Rein et al., 2017), systematic reviews examining the diagnostic accuracy of existing triage tools (van der Sluijs et al., 2018; van Rein et al., 2017, 2018; Voskens et al., 2017), cost-effectiveness evaluations (Newgard et al., 2016; Pollard et al., 2022) and the role of clinical judgement (Newgard et al., 2012; van Rein et al., 2018). This evidence included all relevant systematic reviews available at the time of the consensus meeting, supplemented by the most valid studies identified from ad hoc MEDLINE searches for topics where a pre-existing research synthesis was not available. Participating experts also had the opportunity individually to highlight important supporting evidence.

The meeting then proceeded in a structured format covering the purpose, target reference standard, optimal diagnostic threshold, scope, intended population and structure of the triage tool in repeated rounds of iterative discussions. The findings of the Delphi survey were then re-reviewed, individual triage tool criteria were discussed, and final variables and thresholds were confirmed. Discussions were facilitated to ensure all issues were thoughtfully deliberated, incorporated diverse experience and views, and produced the best possible decision (Halcomb et al., 2008; Jones & Hunter, 1995). Consensus was defined *a priori* as ‘finding a decision together that all members can feel comfortable with’ (Jones & Hunter, 1995). Consensus was developed through group negotiation mediated by the meeting chairpersons.

Third, a further facilitated expert consensus round-table meeting was conducted to confirm a final triage tool, informed by results of the MATTS retrospective validation study. This validation study, reported in detail elsewhere, assessed the diagnostic accuracy of the expert-derived MATTS triage tools and individual triage variables (Fuller, forthcoming). This meeting was held online due to COVID-19 restrictions in July 2021 and was chaired by a single independent clinician (SG), but otherwise followed the same methodology as described above, including the same participants. The meeting focused on any changes to included triage tool variables and cut points. At this meeting detailed definitions were also developed for included variables.

Fourth and finally, following the consensus meeting, separate focus groups were conducted with the Sheffield Emergency Care Forum and Birmingham Injuries PPI groups (Hirst et al., 2016; Then et al., 2014). Focus groups began with a short presentation of the proposed triage tool and, following a series of familiarisation questions designed to build rapport between the interviewer and interviewees, participants were asked to discuss their perspectives. A single moderator led all interviews (GF or JM) and a second researcher was present to take field notes (CH). Sessions continued until code saturation was reached (‘heard it all’). Discussions were audio recorded and later transcribed verbatim by a professional transcriptionist. Analysis followed the Framework Method, with familiarisation, thematic development, indexing, charting and mapping with interpretation (Gale et al., 2013).

## Results

A review of existing NHS ambulance service major trauma triage tools in England identified 97 unique variables grouped into 25 categories across four domains (see Supplementary materials). A further 12 novel variables and two novel categories were suggested by participants in the first round of the Delphi survey (see Supplementary materials). In Round 2, these 109 variables were grouped under 27 categories, across four domains: physiology (three categories), anatomical injury (nine categories), mechanism of injury (nine categories), and special circumstances (six categories). Consensus was achieved on inclusion of two domains and nine categories, and exclusion of seven categories. In Round 3, a further six categories were excluded. At this stage it was apparent that agreement on the remaining categories was declining. It was considered that further sampling would lead to declining response rates rather than more relevant findings, and selection of categories and variables was therefore terminated after Round 3. The variable (and cut point) with the greatest agreement was confirmed for each selected category in each domain at this stage. Table 1 summarises the responses across Delphi Rounds 1 to 3 confirming triage tool domains and categories. Final selected triage tool domains and variables are detailed in Table 2.

**Table 1.** Results for Delphi Rounds 1–3 selecting triage tool domains and categories.

Domain/variable category	Round 2 results		Result	Round 3 results		Result
	Agree	Disagree		Agree	Disagree	
**Physiology domain**	100%	0%	Included	-	-	Included
Level of consciousness	100%	0%	Included	-	-	Included
Circulation	93%	7%	Included	-	-	Included
Ventilation	89%	11%	Included	-	-	Included
**Anatomy domain**	100%	0%	Included	-	-	Included
Chest injury	100%	0%	Included	-	-	Included
Spinal injury	100%	0%	Included	-	-	Included
Pelvic injury	98%	2%	Included	-	-	Included
Long bone injury	98%	2%	Included	-	-	Included
Penetrating injury	96%	4%	Included	-	-	Included
Limb/extremity injury	93%	7%	Included	-	-	Included
Skull fracture	67%	33%	No consensus	37%	63%	Excluded
Burns	67%	33%	No consensus	56%	44%	No consensus
Haemorrhage control	56%	44%	No consensus	37%	63%	Excluded
**Mechanism of injury domain**	73%	27%	No consensus	61%	39%	No consensus
Vehicle ejection	76%	24%	No consensus	61%	39%	No consensus
Falls	73%	27%	No consensus	61%	39%	No consensus
Vehicle entrapment	53%	47%	No consensus	26%	74%	Excluded
Motor vehicle collision	53%	47%	No consensus	32%	68%	Excluded
Blast injury	51%	49%	No consensus	24%	76%	Excluded
Occupant fatality	44%	56%	Excluded	-	-	Excluded
Crush injury	40%	60%	Excluded	-	-	Excluded
Vehicle deformation	29%	71%	Excluded	-	-	Excluded
Assault	11%	79%	Excluded	-	-	Excluded
**Special circumstances domain**	67%	33%	No consensus	58%	42%	No consensus
Pregnancy	67%	33%	No consensus	58%	42%	No consensus
Bleeding risk	60%	40%	No consensus	58%	42%	No consensus
Age	53%	47%	No consensus	45%	55%	Excluded
Co-morbidity	31%	69%	Excluded	-	-	Excluded
Intoxication	22%	78%	Excluded	-	-	Excluded
Patient preference	9%	91%	Excluded	-	-	Excluded

**Table 2.** Evolution of the MATTS triage tool content.

Domain	Delphi survey	First consensus meeting	Second consensus meeting	Comment
**Physiology**	Sustained respiratory rate <10 or >29 Sustained systolic BP<90 or absent radial pulses GCS motor score 4 or less	Sustained respiratory rate <10 or >29* Sustained systolic BP<90 or absent radial pulses* GCS motor score 4 or less	Sustained respiratory rate <10 or >29* Sustained systolic BP<90* GCS motor score 4 or less*	Absent radial pulses omitted to simplify tool at second consensus meeting.
**Anatomy**	Chest injury with altered physiology Suspected major pelvic fracture 2 or more long bone fractures Penetrating injury to neck, chest, abdomen, back or groin Neck or back injury with paralysis Amputation or mangled extremity proximal to ankle or wrist	Chest injury with altered physiology Suspected major pelvic fracture 2 or more long bone fractures Penetrating injury to neck, chest, abdomen, back or groin Neck or back injury with paralysis Amputation or mangled extremity proximal to ankle or wrist Open fracture proximal to forefoot/wrist** Open or depressed skull fracture	Chest injury with new oxygen requirement Major pelvic fracture 2 or more proximal long bone fractures Penetrating injury to neck, chest, abdomen, back or groin Neck or back injury with paralysis Amputation or mangled extremity proximal to ankle or wrist Open fracture proximal to forefoot/wrist** Open or depressed skull fracture	Open fractures and skull fractures added to meet MATTS reference standard definition at first consensus meeting. Chest injury with new oxygen requirement and proximal long bone fractures detailed to increase specificity at second consensus meeting. Suspected pelvis fracture changed to major pelvis fracture at second consensus meeting to improve specificity.
**Clinical judgement**		Significant clinical concern for major trauma requiring MTC care	Significant clinical concern for major trauma requiring MTC care	Clinical judgement domain added to provide established benefit of clinical acumen at first consensus meeting.

*Use JRCALC abnormal paediatric values if <16 year;

**Optionally could be transported to a TU with ortho-plastic capabilities dependent on local network configuration.

An additional Delphi round, examining triage tool implementation, reached consensus on the following categories: inclusion of immediate clinical risk and a 60-minute journey time threshold for bypass. The Delphi survey was terminated due to declining response rates at this point. Consideration of enhanced care team support and inclusion of clinical judgement fell just short of consensus. Table 3 summarises opinions on additional implementation categories and descriptors. Of the 91 experts invited to participate in the survey, 54 completed the first round (59%), with response rates of 45 (49%), 38 (42%) and 32 (35%) achieved in each subsequent round.

**Table 3.** Results for Delphi Round 4 selecting implementation categories and criteria.

For all injured adults who **TRIGGER** the triage tool:			
Implementation category	Level of agreement	Most appropriate descriptor	Level of appropriateness
Prompt consideration of enhanced care team support	**78%**	*Prompt:*	
		Discuss with enhanced care team	2.4
		Request/activate enhanced care team	2.2
		Consider requesting/activating enhanced care team	2.2
		Consider discussing with enhanced care team	1.9
Include consideration of immediate clinical risk	**81%**	*Immediate clinical risk criteria:*	
		Difficulty maintaining airway	3.2
		Imminent cardiac arrest	3.1
		Difficulty controlling haemorrhage	2.9
		Difficulty maintaining oxygenation	2.8
		*Action if any immediate clinical risk criteria triggered:*	
		Triage to nearest trauma unit	2.8
		Request/activate enhanced care team	2.6
		Seek clinical advice	2.4
		Consider requesting/activating enhanced care team	2.2
Include a time to MTC ED arrival threshold	**78%**	*Action if time threshold exceeded:*	
Within 60 minutes’ journey	81%	Seek clinical advice	2.6
Within 90 minutes of 999 call	16%	Consider helicopter to facilitate direct transport to MTC	2.6
Within 45 minutes’ journey	3%	Request/activate enhanced care team	2.4
		Consider requesting/activating enhanced care team	2.4
		Triage to nearest trauma unit	2.3
For injured adults who **DO NOT TRIGGER** the triage tool:			
	Level of agreement	Most appropriate descriptor	Level of appropriateness
Include clinical concern of the healthcare professional on scene	**72%**	*Action:*	
		Seek clinical advice	3.0
		Consider triage to major trauma centre	2.4
		Consider clinical advice	2.2
		Triage to major trauma centre	1.8

Following a review of relevant evidence during the facilitated roundtable expert consensus meeting, iterative rounds of discussion achieved consensus on six aspects of the triage tool: purpose, target reference standard, scope, intended population, structure and optimal diagnostic threshold. In terms of purpose and reference standard, agreement was achieved that the triage tool should be used to identify patients who would benefit from bypassing and pre-alerting to MTCs, rather than injury severity per se, as defined by the previously published MATTS major trauma reference standard (Fuller et al., 2021). This reference standard included the need for time-critical trauma interventions, significant individual injuries requiring expedited specialist management and polytrauma benefitting from early MTC care; it excluded patients where MTC care would not be beneficial (e.g. advanced frailty).

Expert consensus established that the triage tool was intended for use by ambulance service clinicians. Helicopter emergency medical services (HEMS) and pre-hospital doctor services were considered out of scope, as their additional training and experience would allow accurate use of unstructured expert clinical judgement.

Detailed inclusion and exclusion criteria were also agreed. It was recognised that major trauma can occur from low-energy mechanisms of injury (e.g. ground-level falls), and the intended population was therefore confirmed as any patients presenting following a non-trivial injury event. For simplicity, ease of use in the challenging pre-hospital environment and consistency with the NIHR MATTS commissioning brief, all age groups were included in a single triage tool. It was accepted that the triage tool should not be applied in cases where bypass would obviously not be beneficial, or could be harmful, including prolonged journey (>60 minutes travel time) to an MTC, unmanageable airway, breathing or uncontrollable external bleeding, traumatic cardiac arrest with >15 minutes travel time to a MTC, advanced frailty, end of life care or advanced directives. Isolated burns were also excluded due to the existence of separate NHS major burns networks, which may not overlap with major trauma networks. Cases with isolated hypoxic brain injury (e.g. drowning or non-judicial hangings) were also judged ineligible, as it was recognised that these usually lack concomitant physical injuries.

Complete agreement was evident that the triage tool should be structured as a checklist of individual variables assessed in parallel, with variables grouped into physiology and anatomical injury domains to maximise familiarity and concordance with currently used tools. A target trade-off between under- and over-triage was confirmed according to previously published MATTS economic and operational modelling, which indicated prioritisation of specificity for cost-effective major trauma triage (Pollard et al., 2022). The importance of clinical acumen, over and above stated triage variables, was agreed, with an additional domain for unstructured clinical judgement unanimously agreed. Decision support provided by senior clinical staff, operationalised through a trauma/critical care desk, was felt to be important in optimising this clinical gestalt. It was also felt that deployment of enhanced care team support (e.g. HEMS, critical care paramedics, pre-hospital physicians) would be best facilitated via this senior clinical support.

Individual triage variables were defined by consensus, as detailed in Table 2. All variables reaching consensus in the Delphi survey were included in the triage tool, with open fractures and significant skull fractures variables added to ensure conformity with the target MATTS reference standard definition. It was recognised that services may vary regionally, and the option for isolated open fractures to be transported to a TU with ortho-plastic capabilities was allowed, dependent on local trauma network configuration. Two further exploratory triage tools with differing triage variables were also developed for future evaluation, targeting relatively more sensitivity and balanced sensitivity/specificity (Supplementary materials).

**Figure 2:**
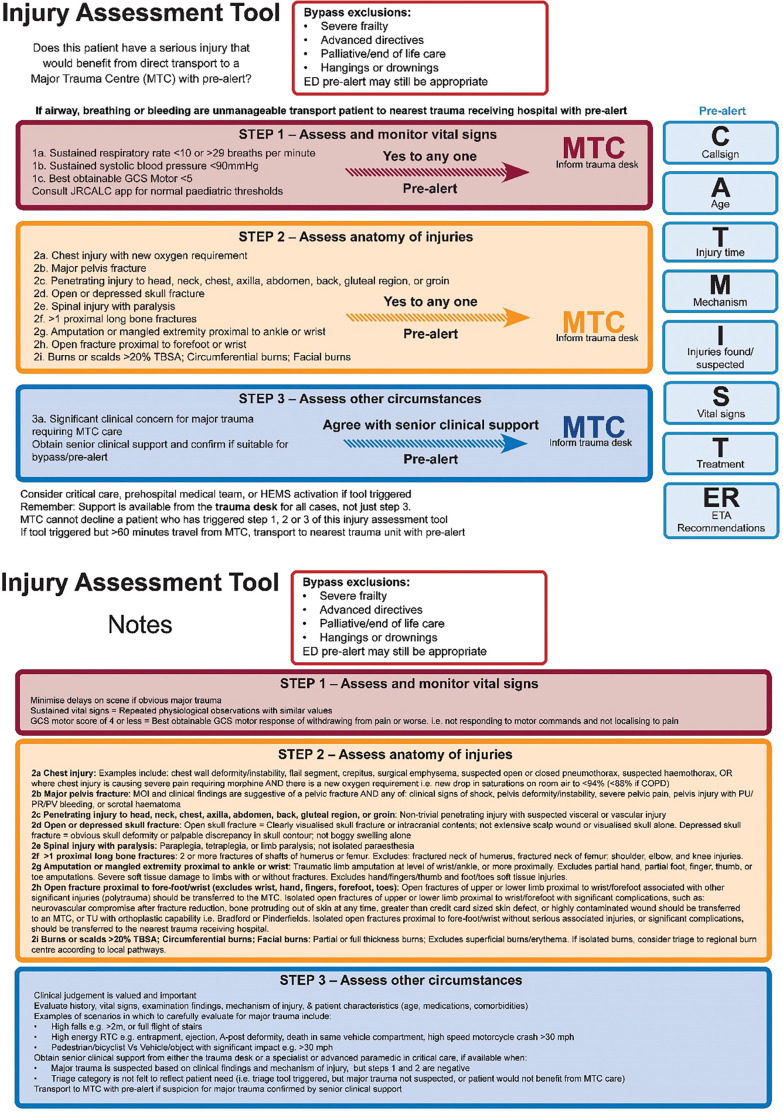
Figure 2. Final MATTS triage tool.

In the second facilitated roundtable meeting, the expert consensus group drew on information from the MATTS retrospective validation study (Fuller, forthcoming) to make minor modifications to the triage tool. Examination of individual triage predictors revealed that absent radial pulses did not provide additional benefit over SBP<90 and this variable was therefore dropped to simplify the tool. Modification of >2 long bone fractures to >2 proximal long bone fractures, and chest injury with altered physiology to chest injury with new oxygen requirement, both appeared to slightly improve triage tool specificity with no loss in sensitivity. No other variables appeared to offer the possibility of improvement in performance. Detailed variable definitions were also confirmed, including a minor change in terminology from ‘suspected major pelvis fracture’ to ‘major pelvis fracture’ to improve specificity. Further results of the retrospective validation study are described in detail elsewhere (Fuller, forthcoming). The final triage tool variables are presented in Table 2 and the final triage tool, including detailed variable definitions, is presented in Figure 2.

Focus groups with the Sheffield Emergency Care Forum and Birmingham Injury PPI groups included 12 and 15 participants, and lasted 63 and 94 minutes, respectively. The triage tool was received favourably, with no negative feedback specific to the triage tool structure, format or variables. Exclusion of bypass in advanced frailty was accepted. A minority of participants voiced strong opinions about patient choice in determining their desired location of care following injury, specifically referencing travelling distances and perceived quality of care in individual hospitals. Representative comments included: ‘Please remember that many older trauma patients will have carers who are also older and can’t travel far’, ‘An MTC probably provides better care for all levels of injury’ and ‘For me, it depends which hospital they’re trying to take me to . . . I would never let them take me to [non-MTC name]’.

## Conclusions

We present a new expert consensus-derived major trauma triage tool for use in English trauma networks, defining purpose, scope, intended population, structure, constituent variables and thresholds. Prospective evaluation is required to determine clinical and cost-effectiveness, acceptability and usability.

Existing English major trauma triage tools were introduced into evolving regional trauma systems between 2010 and 2012, based on the American College of Surgeons Committee on Trauma guidelines for field triage. These trauma systems followed one of two broad conceptual models, often referred to as ‘inclusive’ and ‘exclusive’ (Mackenzie et al., 2014). The models differ in the extent to which available hospitals can provide major trauma care. In ‘exclusive’ systems, the underpinning philosophy is that all patients with suspected major trauma within the area covered by the MTC will be primarily transferred there from the scene, bypassing all other facilities. Exclusive systems are ideally suited for more densely populated urban areas with relatively short pre-hospital journey times. Triage tools designed for exclusive systems aim to minimise under-triage and typically have lower thresholds for bypass (prioritising sensitivity over specificity). Conversely, the underpinning philosophy in ‘inclusive’ systems is that, by virtue of geography, longer pre-hospital journey times, MTC capacity constraints and system efficiency, TUs must be able to receive and transfer major trauma patients. Triage tools designed for inclusive systems aim to minimise over-triage and typically have higher thresholds for bypass (prioritising specificity over sensitivity).

A recent document analysis identified 53 English, other United Kingdom (UK) and international major trauma triage tools, begging the question of why another triage tool is required (Fuller, forthcoming). However, the use of a different tool in each English ambulance service and observed wide variation of structure and content, combined with unsatisfactory performance reported in systematic reviews examining diagnostic accuracy, suggests there is a need for development of a new standardised major trauma triage tool for the NHS. This expert consensus process was designed to build on previous work, rather than develop a radically new triage tool, offering the opportunity to address deficiencies in previous triage tool development and directly address the English context. The MATTS project has also retrospectively validated existing tools, together with the proposed tool reported herein, allowing selection of an optimally performing tool (Fuller, forthcoming).

There are several potential methods for developing a triage tool, including expert consensus (Murphy et al., 1998), statistical modelling (Steyerberg, 2009) and machine learning (Hastie et al., 2009). There are many grounds for supporting the methodological choice of using an expert consensus process. First, paper-based expert-derived tools, structured as checklists of variables, are intuitive and simple to use in the challenging pre-hospital environment. Second, such a design is currently used by all English ambulance services, reducing the risk of implementing a similarly designed national tool, allowing learning from current experience, and offering the opportunity to modify, rather than overhaul, existing tools. Third, qualitative research has highlighted that paper-based ‘checklists’ are highly valued by pre-hospital clinicians (Fuller, forthcoming). Fourth, expert-derived clinical decision rules have been demonstrated to perform well across many disease areas (e.g. the HEART score in chest pain evaluation) (Six et al., 2008). Fifth, statistical prediction models and machine-learning algorithms have increased complexity, often require very large robust datasets for development, may be less interpretable and are often difficult to implement into clinical practice (Andaur Navarro et al., 2023; Bouwmeester et al., 2012; Volovici et al., 2022). Methodology guidelines therefore advise that existing clinical decision rules should be refined and validated where possible (Stiell & Wells, 1999). Finally, machine learning would be expected to poorly perform *ex ante* in low signal to noise situations, as might be expected in major trauma triage (Hastie et al., 2009).

Our understanding of the epidemiology of serious injury has evolved over recent decades, with an increased incidence of low-energy ‘silver trauma’ (Coats & Lecky, 2017). Major trauma could be considered as different disease entities in children, adults and older people, with each demonstrating differing mechanisms of injury, injury patterns and physiological responses. The MATTS project commissioning brief was to develop a single triage tool; however, it could be argued that a ‘one size fits all’ approach is unlikely to perform well across all injury subgroups and geographic populations. Conversely, using multiple different triage tools might be impractical in the pressured pre-hospital setting. Prediction of major trauma in older people may also be difficult, with reduced accuracy demonstrated for vital signs, for example, limiting the potential gain from bespoke triage tools (Pandor et al., 2022). Given the heterogeneity of major trauma, differential triage tool accuracy should be expected across age subgroups, blunt/penetrating and high/low-energy trauma and different anatomical injuries. Moreover, performance should not be considered as static, and tool updating will be required in the future as new evidence emerges and if injury profiles change.

A vital first triage tool design step is defining the reference standard of which injuries benefit from bypassing and/or pre-alerting to MTCs. A second essential consideration is characterising a threshold for decision-making that optimises the trade-off between false positives and false negatives. The American College of Surgeons Committee on Trauma has recommended an under-triage fraction of ≤5% (i.e. sensitivity of >95%) and over-triage fraction of ≤35% (i.e. specificity of >65%) (Newgard et al., 2022). However, it is important to consider the benefits, harms and costs of prioritisation; economic evaluations have consistently demonstrated that a high-sensitivity triage approach consistent with such benchmarks is not cost-effective, and in contrast specificity should be emphasised (Maughan et al., 2022; Newgard et al., 2016; Nishijima et al., 2021; Pollard et al., 2022). Interestingly, previous English triage tools have not explicitly stated their target definition of major trauma or desired under/over-triage trade-off. English trauma networks are commissioned as inclusive systems, providing further *a priori* rationale for targeting higher specificity (NHS England, 2013).

The population for which the triage tool is intended also requires careful description. Where defined, the intended patient population for application of current English triage tools is high-energy trauma or suspicion of major trauma (Fuller, forthcoming). However, ‘stealth trauma’ is an increasingly recognised phenomenon, with a significant proportion of severe injuries occurring with low-energy mechanisms or presenting atypically (Davies & Coats, 2020). This could support conceptualisation of the major trauma triage tool as an ‘injury assessment tool’ to maintain user familiarity and avoid missing assessment of seriously injured patients. Considering any barriers to triage tool application is also important for interpretation of triage tool performance. Triage tools will demonstrate spectrum effects, with performance varying at different pre-test probabilities of major trauma (Usher-Smith et al., 2016). Moreover, positive predictive value will tend to be lower if applied to low prevalence populations, and negative predictive value will be reduced if used in higher prevalence populations. A Bayesian approach of formulating a pre-test probability of major trauma based on mechanism of injury, with application of corresponding likelihood ratios following application of the triage tool, and consequent calculation of post-test probability, is therefore recommended (Florkowski, 2008).

A recent document analysis of existing triage tools identified an array of expert-derived triage tools, with three distinct patterns: ‘specific and prescriptive’, ‘intermediate’ and ‘sensitive and permissive’ (Fuller, forthcoming). The final recommended MATTS triage tool has similar variables and cut points to the ‘specific and prescriptive’ grouping, but also adds an element of unstructured clinical judgement. Detailed variable descriptions are provided to reduce ambiguity. Major trauma triage tools are commonly deployed within a complex milieu, potentially involving overlapping ambulance service, major trauma network and specialist services catchment areas (e.g. burns, ortho-plastics). Multiple pre-hospital resources may be available to respond to suspected major trauma cases, including helicopter emergency medical service (HEMS), pre-hospital physician-paramedic teams or specialist critical care paramedics. Major trauma specialist services may also be spread across different hospitals, within or across NHS trusts/healthcare organisations. Moreover, hospital TU and MTC designations may differ depending on paediatric or adult presentations. To operate effectively, major trauma triage tools may therefore need to provide additional context and instructions beyond a checklist of triage variables, including exclusion criteria, injury-dependent destination advice and the option for senior clinical support, for example from a dedicated ambulance service ‘trauma/critical care desk’, to navigate these intricacies.

This study has several limitations. External validity to non-UK settings is questionable due to differences in trauma system configuration, health service organisation, patient values and accepted clinical standards of care. Moreover, it is possible that individual views within the expert panel may not be fully consistent with evidence-based triage practice or could be unrepresentative of general clinical opinion. The final tool was also not reviewed by pre-hospital clinician end users, although a detailed qualitative evaluation was conducted later after implementation of the tool into practice. In contrast, the strengths include a comprehensive sample of pre-hospital, MTC and non-MTC experts, PPI input and conformity with consensus study guidelines (Halcomb et al., 2008; Jones & Hunter, 1995). The inclusion of local experts should ensure this triage tool is directly applicable to the English NHS. Other devolved countries within the UK have introduced similar trauma networks to England, and have comparable national health services, suggesting good generalisability to these settings. The consensus approach allowed fuller deliberation of the decision problem, compared to other decision-making approaches, such as executive decisions or majority rule. Roundtable methodology is better suited to complex, multi-dimension constructs than other consensus methodology, such as the Delphi process or nominal group technique, which are designed to answer narrow, single-issue questions (Halcomb et al., 2008; Jones & Hunter, 1995).

There are several implications for further research and clinical practice arising from this research. The proposed MATTS triage tool requires prospective validation and evaluation in a representative English setting. Confirmation of diagnostic accuracy, cost-effectiveness, acceptability to ambulance services and trauma networks, and safety is required prior to any recommendation for national use. Only variables from clinical examination were included, and future research examining novel point of care testing, such as lactate or ultrasound, might provide additional utility. Evaluation of integration of the triage tool into electronic patient records, or implementation as an electronic application, would also be informative.

## Acknowledgements

We would like to acknowledge members of the MATTS triage tool consensus panel for their help and expertise: Nicola Batrick, Mark Faulkner, Nathan Howes, Rob Jones, Anthony Kehoe, Allie Klein, Josh Miller, Chris Press, Stuart Reid, Simon Standen and Paul Younger. We are also very grateful to the Sheffield Emergency Care Forum and Birmingham Injury PPI groups for their help and support. This research was supported by the MATTS study management group, including: James Baird, Richard Pilbery, Natalie Kean, Fiona Lecky, Antoinette Edwards, Ian Maconochie, Mathew Ward, Mark Millins, Emily Turton, Simon Waterhouse, Matt Stevenson, Daniel Pollard, Abdullah Pandor, Maria Robinson, Stuart Reid, Di Charles, Andy Rosser, Rachael Fothergill, Sarah Black, Fiona Bell, Michael Smyth, Jason Smith, Gavin Perkins, Esther Herbert, Stephen Walters and Cindy Cooper.

## Author contributions

GF, NH and RM conceived the study. GF, NH and SK managed the study. NH and RM performed and managed the Delphi study. JS and SG co-chaired the consensus meeting. The MATTS triage tool panel developed the definition of injury benefitting from expedited MTC care. JM and CH performed the PPI focus groups. All authors made substantial contributions to the design, data processing and interpretation. All authors had full access to all the data in the study and can take responsibility for the integrity of the data and the accuracy of the data analysis. JT acts as the guarantor for this article.

## Conflict of interest

None declared.

## Ethics

Ethical approval was provided by Yorkshire and The Humber ‒ Bradford Leeds Research Ethics Committee (Reference: 19/YH/0197). All participants provided informed consent.

## Funding

The study was funded by the National Institute of Health Research Health Technology Assessment Programme (Grant: 17/16/04) as part of the larger MATTS project.
